# Unveiling the gap in heart failure: a Brazilian Unified Health System study

**DOI:** 10.7189/jogh.16.04097

**Published:** 2026-05-15

**Authors:** Nathalia Volpi e Silva, Vicky Nogueira-Pileggi, Renato Lima Vitorasso, Daniela Vicentini, Elizabeth Bilevicius, Vivian Cardoso Batista, Renato Mantelli Picoli

**Affiliations:** 1 Oracle Life Sciences, São Paulo, Brazil; 2 Research, Development and Medical Affairs, Viatris, São Paulo, Brazil

## Abstract

**Background:**

Heart failure (HF) decompensation is the leading cause of hospitalisations in developed countries and the third most common cause in Brazil. Underdiagnosis or misdiagnosis thereof remains a critical challenge and with significant implications. At the patient level, it delays appropriate treatment and disease management. At the health system level, it leads to inefficient population health strategies, distorted epidemiological estimates, and suboptimal resource allocation. Addressing this hidden HF population is therefore essential for improving disease surveillance, optimising care pathways, and supporting more effective and equitable health system planning in Brazil. In this sense, we aimed to quantify underreporting of HF in individuals in Brazil and to estimate their mortality trends.

**Methods:**

We identified potential HF patients using a guideline-based flowchart that combines ICD-10 codes (*e.g.* I21, I25, I42) and HF-related procedures (*e.g.* echocardiography, B-type Natriuretic Peptide (BNP) testing). Then, we developed a machine learning model for early prediction of HF using the Brazilian Department of Informatics of the Unified Health System (*Departamento de Informática do Sistema Único de Saúde*) mortality information system and ambulatory information system records, 2018–2022. A CatBoost algorithm was trained on a balanced cohort of patients (10 000 with HF and 9 995 without HF), restricting predictors to ≥12 months before the first I50 code to prevent leakage. Key predictors included age, chronic kidney disease, echocardiography, and lipoprotein disorders. We validated model performance in independent cohorts, including a low-prevalence cohort (~2% HF). Finally, we derived underreporting estimates via deterministic sensitivity analysis across scenarios from 0–100%.

**Results:**

The proxy identified approximately 54 000 potential HF cases/y that were unreported in ambulatory data, with deterministic analysis suggesting 12–41% underestimation. Mortality analysis showed that around 200 000 deaths could be linked to unreported HF. The CatBoost model achieved an area under the curve (AUC) of 0.91 (balanced cohort; accuracy = 0.82, recall = 0.81, F1 = 0.82) and maintained strong discrimination in low-prevalence settings (AUC = 0.84, sensitivity = 0.82), with excellent calibration (Brier = 0.124–0.136; ECE = 0.01–0.03).

**Conclusions:**

We found that HF underreporting in Brazil is substantial and carries significant mortality implications. Our ML model demonstrates high accuracy in early risk stratification using routine administrative data, aligning with clinical pathways. Implementing it as a screening tool could optimise resource allocation, but ethical considerations around false positives warrant careful deployment. Future work should focus on clinical validation and cost-effectiveness analysis within the Brazilian Unified Health System. Furthermore, our findings highlight significant implications for public health and clinical HF management, emphasising the necessity of strategies that promote early detection of HF and precise case recording. Addressing underestimation is crucial to optimise healthcare resources and improve patient outcomes. The results underscore the importance of accurate diagnosis and comprehensive management approaches for better HF case tracking. Further research should explore the public health impact of these underestimations in Brazil, particularly regarding its health system’s financial resources.

According to the World Health Organization (WHO), cardiovascular diseases (CVDs) are a leading cause of death worldwide, despite being preventable through behavioural and clinical interventions. They accounted for around 17.9 million deaths in 2019, representing 32% of all global deaths [1].

Heart failure (HF) stands out as a highly prevalent CVD, associated with significant morbidity, mortality, and healthcare costs. It has been estimated that HF alone affected 23 million people worldwide in 2017, and its prevalence is projected to increase by around 46% by 2030 [2]. The condition is clinically defined as a syndrome with symptoms and/or signs caused by structural and/or functional cardiac abnormality, corroborated by elevated natriuretic peptides and/or objective evidence of pulmonary or systemic congestion [3].

In Brazil, HF imposes a particularly heavy burden on the public health system. It was the main cause of CVD hospitalisations between 2008 and 2018, with more than two million hospitalisations having been recorded and more than 567 000 deaths in adults aged over 50 years [2]. The risk of developing HF increases with age, and although the prevalence is higher among the elderly, there is a growing number of cases in individuals in the 50-year-old group. In 2019 alone, HF-related hospitalisations and mortality rates were responsible for health services expenses that exceeded three billion Brazilian Reais (BRL) (USD 626.5 million) [2]. HF decompensation is the main cause of hospitalisations in developed countries, and in Brazil, it represents the third leading cause [4].

According to the Brazilian Chronic Heart Failure Treatment Guideline, patients with HF have a worse prognosis compared to the general population, with no substantial difference in global mortality among HF subtypes [5]. There is also a higher risk of mortality during the first year after an HF diagnosis, as the condition is frequently identified only once patients develop acute or advanced symptoms [5]. The absolute number of deaths has remained stable over the last decade and more than 50% of HF patients experience sudden deaths [6].

Despite this substantial burden, the diagnosis of HF in routine clinical practice remains challenging. This is partly due to the presence of multiple comorbid diseases, such as chronic obstructive pulmonary disease, ischaemic heart disease, and chronic kidney disease, that share overlapping symptoms and may act as diagnostic confounders, especially when HF presents with mild symptoms or non-specific manifestations [5,6]. In addition, in administrative databases such as the Brazilian Department of Informatics of the Unified Health System (*Departamento de Informática do Sistema Único de Saúde* (*DATASUS*)), diagnostic codes are often assigned to authorise procedures or reflect a working diagnosis, which may not always correspond to a confirmed HF diagnosis. As a result, the true prevalence and mortality burden of HF in Brazil may be underestimated in official statistics.

Large administrative databases such as *DATASUS* offer a unique opportunity to monitor the care trajectory of millions of patients across the Brazilian Unified Health System (*SUS*). However, these databases were not originally designed for epidemiological surveillance or early detection: they lack detailed clinical measurements (*e.g.* laboratory values, imaging results) and rely heavily on ICD-10 codes and procedure records. Traditional epidemiological approaches may not fully capture complex, nonlinear patterns that precede an HF diagnosis in such noisy and incomplete data.

At the health-system level, this leads to inefficient population health strategies, distorted epidemiological estimates, and suboptimal resource allocation. Consequently, under- and misdiagnosed HF patients may contribute disproportionately to avoidable hospitalisations, emergency visits, and overall healthcare expenditures due to irrational or misusedhealthcare resources. Therefore, understanding the real impact of the HF on the Brazilian population and addressing this hidden HF population is essential to improve disease surveillance, optimise care pathways, and support more effective and equitable health system planning in Brazil.

Machine learning (ML) algorithms are particularly well-suited to this challenge, as they can process high-dimensional information from longitudinal administrative records and identify subtle combinations of diagnoses, procedures, and demographic factors that are associated with future HF, even when HF has not yet been formally coded. By learning from patterns observed in patients who eventually receive an HF diagnosis, ML models can be used to assign a risk score to individuals who have not yet been diagnosed, potentially identifying patients at risk months or years before clinical recognition. In the Brazilian context, where underdiagnosis and misclassification are concerns and resources are limited, such models may support clinicians and health managers in prioritizing high-risk patients for further evaluation and optimising resource allocation.

In light of these considerations, we aimed to identify potential patients with HF in the Ambulatory Information System of the Brazilian Unified Health System (*Sistema de Informações Ambulatoriais do Sistema Único de Saúde* (*SIA-SUS*)) and provide an estimate of the underreporting of HF in Brazil, and to describe the estimated mortality of potential HF patients in Brazil.

## METHODS

We first defined a clinically grounded flowchart for identifying potential HF patients in the ambulatory care setting, using a combination of ICD-10 codes and key diagnostic and therapeutic procedures recorded in the *SIA-SUS* system. This flowchart was based on Brazilian HF guidelines and on conditions known to be strongly associated with HF development or evaluation, thereby providing a proxy to estimate the underreporting of HF in *DATASUS*. Here, we operationally defined underdiagnosis as the presence of patients whose clinical profile and care trajectories are highly compatible with HF, but who never receive an I50 code in *DATASUS*, and misclassification as HF-related deaths and hospitalisations that are coded under other cardiovascular diagnoses (*e.g.* ischaemic heart disease, myocardial infarction). Our work addresses a primarily methodological and epidemiological gap: how to use routinely collected administrative data to uncover this ‘hidden’ burden of HF in Brazil and to estimate its impact on mortality.

Second, we developed and validated a ML predictive model, using the CatBoost algorithm, to identify patients at high risk of HF prior to the first formal I50 code in *DATASUS*.

### Clinical flowchart for HF and descriptive analyses

We first sought to determine how patients with HF go through Brazil’s health system. In this study, we employed data from *DATASUS*, which encompasses information from the SUS. Specifically, we utilised the Mortality Information System of the Brazilian Unified Health System (*Sistema de Informações sobre Mortalidade do Sistema Único de Saúde* (*SIM-SUS*)) and *SIA-SUS*. The data from these systems is accessible to the public via file transfer protocol in .dbc format, while we converted the *SIA-SUS* to a .parquet format. We conducted both data extraction and variable calculations in *R*, version 4.2.0 (R Core Team, Vienna, Austria) and Python, version 3.10.6 (Python Software Foundation Core Developers, Wilmington, DE, USA).

**Figure 1:**
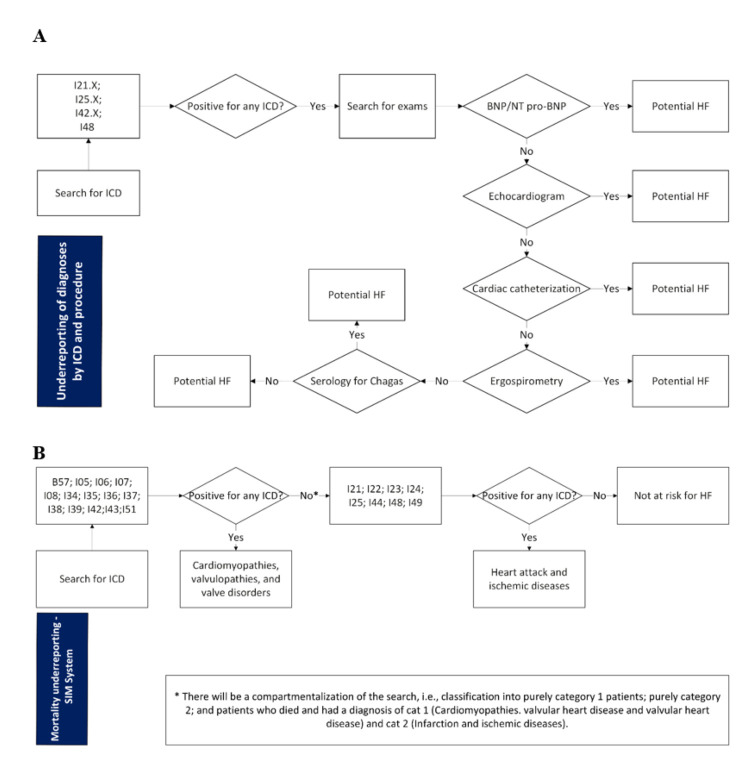
Flowcharts to find underreported patients by ICD and procedure. **Panel A. **Flowcharts from Ambulatory Information System (*SIA-SUS*). **Panel B. **Flowcharts from Mortality Information System (*SIM-SUS*).

Below we present flowcharts delineating the inclusion criteria for the subgroups under study (**Figure 1**), that were developed according to cardiology specialists regarding patient journey and guided by the Brazilian Heart Failure Guidelines [5]. This ensured that the established criteria (Table S1 in the **Online Supplementary Document**), demographic characteristics, ICD-10 codes and medical procedures used at each step of the ambulatory and mortality flowcharts align with Brazil’s clinical and epidemiological realities. Patients who met these criteria, but had no I50 code recorded in *SIA-SUS* (for ambulatory data) or on the death certificate (for *SIM-SUS*) were classified as potential HF cases or potential HF-related deaths, respectively.

The inclusion criteria varied according to the group studied. We included data from 2018 to 2022; however, since the patient may have had their HF record or potential HF classification prior to 2018, we included three previous years in the extraction to obtain the cut-off without a bias towards the classification of the first years. We included patients who had at least one record of ICD I50 in any ambulatory procedure authorisation of *SIA-SUS* and classified them as patients with HF; deaths of patients who had an ICD I50 note on the death certificate in the base cause, or lines A, B, C, D and II, classified as HF-related deaths; patients who complied with the flowchart to find underreported patients by ICD and procedure from *SIA-SUS* (**Figure 1**, Panel A) and did not have an ICD I50 record, classified as potential patients with HF; and patients who complied with the flowchart to find underreported patients from *SIM-SUS* **(****Figure 1**, Panel B) and did not have an ICD I50 record on the certificate were identified as potential deaths related to HF. We searched the indicated ICDs in all available fields on the death certificate, except for the underlying cause. Additionally, the cause of death had the following subclassifications: cardiomyopathies, valvular heart disease; and heart attack and ischaemic diseases.

We conducted a deterministic sensitivity analysis to assess the rate of underestimation in ambulatory diagnoses and deaths related to HF over the study period. We evaluated best-case, worst-case, and intermediate scenarios to understand the potential variability in the outcomes, providing insights into how different assumptions could impact the estimation of HF-related diagnoses and mortality rates. We calculated the underestimation rate using the following equation and applied it to both the number of cases and deaths:



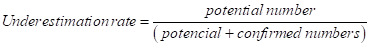



We considered values ranging from 0% (best-case scenario) to 100% (worst-case scenario), *e.g.* 0%, 20%, 40%, 60%, 80%, and 100%, as the proportion of potential patients or deaths correctly identified as actual patients or deaths related to HF. For instance, when determining the underestimation rate for deaths, assuming 20% of potential patients are correctly identified as actual HF patients, the calculation follows this equation:



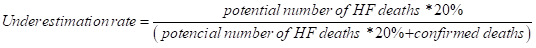



This approach facilitates a thorough investigation into the influence of different proportions of potential patients accurately classified as HF patients on underestimation rates.

We did not intend to designate the 0% and 100% scenarios as realistic estimates; rather, we used them as conceptual bounds to contextualise more plausible intermediate scenarios (*e.g.* 20–60%).

### Development of a ML predictive model for early detection of HF

#### Data source and structure (*DATASUS*/*SIA-SUS*)

We obtained ambulatory healthcare data from the *DATASUS*, specifically, the *SIA-SUS*. This database compiles records of outpatient procedures, laboratory test requisitions, imaging exam requests, consultations, and medication authorisations performed within the public SUS. Importantly, *SIA-SUS* does not contain laboratory or imaging results. Each record includes only information about the procedure, test, or consultation performed or requested; the date of the event; and the ICD-10 code entered by the clinician to justify the service.

In the Brazilian public system, ICD-10 codes in ambulatory records frequently reflect clinical suspicion, rather than confirmed diagnoses, because a diagnostic code must be provided for authorisation of most procedures. As a result, ICD codes in *SIA-SUS* may represent symptoms, suspected conditions, early manifestations of disease, or investigation-related hypotheses rather than established diagnoses.

#### Data preprocessing

To construct the modelling cohort, we first applied mandatory data completeness filters, excluding individuals missing age, sex, weight, height, or race information, as these variables were essential for cohort definition and model development.

For the remaining variables, we designed a structured preprocessing pipeline. We screened continuous variables (*e.g.* age, body mass index (BMI), counts of diagnostic or procedural codes) for implausible values using clinical plausibility thresholds and empirical distribution analysis. Then, we classified values outside physiologic limits (*e.g.* age <18 or >110 years; BMI<12 or >60 kg/m^2^) or extreme outliers above the 99th percentile as probable coding or data-entry errors and removed them from further analysis. We selected the 99th percentile threshold because values beyond this point reflected clear data-entry inconsistencies in *SIA-SUS* (*e.g.* implausible exam counts), as confirmed by manual inspection of their raw frequencies.

After this initial cleaning, continuous variables with missing values were imputed using the median and categorical variables using the mode, both estimated from the training data set to avoid information leakage. We converted categorical variables into binary indicators using label encoding or one-hot encoding, depending on the model requirements. For non-tree-based models such as logistic regression and Support Vector Machine (SVM), we standardised continuous variables using a StandardScaler. CatBoost, which natively handles categorical variables and is robust to monotonic transformations, used raw cleaned values without scaling.

#### Data set construction and feature engineering

We aggregated all *SIA-SUS* records from 2018 to 2022 to generate a patient-level data set suitable for ML. For all participants, we computed the total number of occurrences of ICD-10 chapters and specific three-digit ICD codes; key diagnostic and laboratory test requests (*e.g.* echocardiography, BNP/NT-proBNP, electrocardiogram, ambulatory blood pressure monitoring, *Trypanosoma cruzi* IgG); outpatient cardiovascular-related procedures; demographic characteristics (age, sex, race/ethnicity, age groups); anthropometric measures (height, weight, BMI). This allowed us to reconstruct each patient’s longitudinal clinical trajectory – which is not accessible to clinicians in real time within SUS – enabling the model to identify patterns such as recurrent symptoms, escalating diagnostic investigations, or clusters of comorbidities suggestive of progressive disease, subclinical HF, or underdiagnosis. Count-based features were left untransformed, as tree-based models like CatBoost handle high-cardinality count distributions robustly. Imputation procedures and encoding strategies followed the same approach described in the preprocessing section.

#### Temporal windowing and prevention of data leakage

Because ICD-10 codes in ambulatory records may appear during the diagnostic investigation period, there is substantial risk that pre-diagnosis records could reflect imminent labelling or workup. To mitigate this and ensure that only genuinely pre-diagnostic information informed the model, we applied strict temporal constraints: for all individuals diagnosed with HF in 2022, only events occurring ≥12 months prior to the first recorded I50 code were included. All events within the 12-month pre-diagnosis window were removed to avoid contamination by diagnostic workup, symptom escalation, or pre-labelling. We used no records from 2022 for feature construction, and we used only data from 2018–2021 as model inputs. This design ensured that the model learned from true pre-diagnostic trajectories rather than artefacts of the diagnostic pathway.

#### Cohort assembly and model development

After quality filtering and temporal restrictions, we included 19 995 individuals in the primary modelling cohort: 10 000 patients diagnosed with HF in 2022 (after exclusion of all pre-diagnostic records) and 9995 individuals with no record of HF prior to or during 2022. We constructed the balanced cohort prior to modelling by selecting an equal number of HF and non-HF individuals; however, we applied no additional rebalancing or resampling technique (*e.g.* synthetic minority over-sampling technique, under sampling) within the cross-validation folds, ensuring that all model evaluations reflected the natural class distribution of the sampled cohort.

Once we defined the primary modelling cohort (used for training and internal testing), all its individuals were excluded from the pool of eligible patients for validation. We then sampled the two independent validation cohorts (20 000 and 17 041 patients) from the remaining *DATASUS* population, applying the same inclusion criteria but enforcing patient-level non-overlap. Thus, no individual contributed data to both the development cohort and any validation cohort.

To avoid data leakage, we performed all preprocessing steps (outlier removal, imputation, encoding, and scaling for non–tree-based models) exclusively within each training fold, using parameters learned from the fold-specific training subset. The data set was then split into 80% training and 20% testing using stratified random sampling to maintain the HF/non-HF class distribution. After hyperparameter optimization and selection of the best-performing model, the entire cohort was used for final model training.

To assess external generalisability, we applied the final model to two independent, non-overlapping validation cohorts (n = 20 000 and n = 17 041) drawn from *DATASUS*. We evaluated seven machine-learning algorithms as follows: logistic regression, random forest, gradient boosting, SVM, CatBoost, XGBoost, and AdaBoost. Performance was compared based on the area under the curve (AUC), accuracy, sensitivity, specificity, F1-score, calibration metrics, and confusion matrices.

#### Feature selection

We defined the initial feature set *a priori* based on clinical reasoning, guideline-based evidence, and expert consultation with cardiologists, while using the Brazilian Heart Failure Guidelines as reference [5]. Selected features included demographic variables (age, sex, race, BMI); ICD-10 chapters and three-digit codes associated with HF risk, aetiologies, or comorbidities (*e.g.* ischaemic heart disease, hypertension, Chagas disease, diabetes); diagnostic and therapeutic procedures routinely used in HF investigation (Table S1 in the **Online Supplementary Document**).

Furthermore, we conducted exploratory univariate analysis and data-quality screening to refine the set. We removed variables with extremely low prevalence or near-zero variance. We consolidated ICD codes and procedures, conveying redundant or overlapping clinical meaning by retaining the most clinically informative representation, thereby reducing redundancy and minimising collinearity while preserving interpretability. We intentionally avoided automated wrapper-based feature selection methods (*e.g.* stepwise regression, recursive feature elimination) to maintain a parsimonious and clinically coherent feature set grounded in domain expertise rather than automated heuristics.

#### Model optimisation

For hyperparameter optimisation, we employed ‘randomsearch’ algorithm [7] in conjunction with the ’Scikit-Optimize’ (skopt) [8] library. This approach enabled efficient hyperparameter tuning by exploring a broad range of possible parameter values. We first used ‘randomsearch’ algorithm to identify the best cross-validation-performing hyperparameters for the algorithm that demonstrated the highest performance during the initial screening. The parameter grid used in ‘randomsearch’ algorithm encompassed the following hyperparameters: depth ranging from 1 to 10; learning_rate ranging from 0.01 to 1; iterations ranging from 100 to 1000; l2_lef_reg ranging from 2 to 100; and border_count ranging from 10 to 35.

‘Skopt’, which utilises Bayesian optimisation, further refined the optimisation process by narrowing the search space, improving the efficiency of hyperparameter selection. The hyperparameter space explored during optimisation was defined as follows: depth: integer values ranging from 5.0 to 10.0; learning_rate: real values between 0.01 to 0.5, sampled on a log-uniform scale; iterations: integer values ranging from 500 to 1000; l2_leaf_reg: real values between 2.0 and 35.0, sampled on a log-uniform scale; and border_count: integer values between 10 and 35.

We used stratified 5-fold cross-validation (CV = 5) in all tests to ensure robust and consistent evaluation of the models' performance (Figure S1 in the **Online Supplementary Document**). We evaluated the results of the optimised models based on their predictive receiver operating characteristic (ROC) curve and corresponding AUC, accuracy, recall, and F1-score and generalisation capability using the test data set. Additionally, a partial dependence plot (PDP) of the objective function was generated to visualise the impact of each hyperparameter on the objective function during the optimisation process. We also analysed the learning rate curve to assess the impact of learning rate adjustments on model performance, ensuring that the model was optimised effectively without overfitting or underfitting the data.

We conducted hyperparameter optimisation using a two-stage strategy which combines ‘randomsearch’ algorithm and Bayesian optimisation, implemented through the ‘skopt’ library. In the initial stage, we used ‘randomsearch’ algorithm to explore a broad hyperparameter space and identify configurations yielding superior cross-validation performance for the selected algorithm. The initial search grid comprised the following hyperparameters: depet (1–10), learning_rate (0.01–1), iterations (100–1000), l2_leaf_reg (2–100), border_count (10–35)

Subsequently, we applied Bayesian optimisation to refine the search by leveraging prior information on model performance and narrowing the hyperparameter space. The optimised parameter space included depth (depth (5–10, integer), learning_rate (0.01–0.5, log-uniform continuous), iterations (500–1000, integer), l2_leaf_reg (2–35, log-uniform continuous), and border_count (10–35, integer).

All optimisation procedures employed 5-fold cross-validation (CV = 5) to ensure robust model evaluation. We assessed the optimised models using discrimination metrics (ROC and corresponding AUC) and classification measures (accuracy, recall, F1-score), as well as generalisation performance in the independent test data set. We generated a PDP of the objective function to visualise the marginal effect of hyperparameters on model performance. We also examined learning rate progression to verify the adequacy of learning dynamics and mitigate risks of overfitting or underfitting.

In addition to tree-based and ensemble methods, we trained a regularised logistic regression model using the same feature set and training/validation splits as CatBoost. L2-penalised logistic regression with class-balanced weights and standardised predictors was implemented using scikit-learn. This model served as a baseline to quantify the incremental value of more flexible ML algorithms.

#### Model validation and performance evaluation

We evaluated the performance of the optimised ML model using a comprehensive set of discrimination and classification metrics, in accordance with best practices for clinical prediction modelling in cardiovascular research [5,6]. Discrimination was quantified using the ROC curve and corresponding AUC. Additional performance metrics, including accuracy, recall (sensitivity), and F1-score, were computed to provide a multidimensional assessment of model behaviour. Accuracy represented the proportion of correct classifications; recall assessed the capacity to correctly identify true HF cases; and the F1-score, the harmonic mean of precision and recall, offered insight into classification balance and robustness. We evaluated these metrics on the training and testing data sets, as well as on an independent validation cohort to assess model generalisability.

Furthermore, we conducted a learning curve analysis to characterise the relationship between training sample size and model performance, allowing assessment of potential overfitting or underfitting. We generated confusion matrices for the training, testing, and validation data sets to provide a detailed evaluation of classification performance, including the distribution of true positives, false positives, true negatives, and false negatives.

Given the low prevalence of HF in real-world populations, an additional non-overlapping DATAUS validation, we constructed a subset to reflect epidemiological HF prevalence estimates (~ 2%). All non-HF individuals from the validation data set (n = 8541) were included, and 175 HF cases were randomly sampled, resulting in a prevalence of 1.98%. We used this subset strictly for model evaluation. The model trained on the balanced data set (10 000 HF and 10 000 non-HF) was applied to this population without retraining or recalibration. We calculated standard clinical performance metrics, including AUC, sensitivity, specificity, positive predictive value, negative predictive value, and Brier score, to evaluate performance in this low-prevalence context.

#### Calibration assessment

We conducted a comprehensive calibration assessment to evaluate the reliability of predicted probabilities, an essential requirement for clinical applicability in cardiology, particularly when models are intended to support risk stratification. Calibration was assessed using multiple complementary metrics:

− Brier score: a proper scoring rule quantifying the mean squared difference between predicted probabilities and observed outcomes (range: 0–1; lower values indicate better calibration). Values <0.15 were interpreted as indicative of excellent calibration.
− Hosmer-Lemeshow test: a goodness-of-fit test comparing observed and expected event rates across deciles of predicted risk. A non-significant *P* value (>0.05) was interpreted as evidence of acceptable calibration.
− Expected calibration error (ECE): the weighted mean absolute difference between predicted and observed event frequencies across bins. Values <0.05 reflected high calibration accuracy.
− Maximum calibration error (MCE): the maximum absolute deviation across bins, used to identify regions with the poorest calibration.
− Calibration slope and intercept: Derived from logistic regression of outcomes on logit-transformed predictions. A slope of 1.0 and an intercept of 0.0 indicated ideal calibration.

We generated calibration curves by grouping predicted probabilities into deciles and plotting observed HF event rates against mean predicted probabilities. The 45-degree line represented perfect calibration. Additionally, we examined the distribution of predicted probabilities for HF and non-HF groups (Figure S2 in the **Online Supplementary Document**) to assess model confidence and detect potential miscalibration at probability extremes.

We conducted all calibration analyses in the training data set and in both independent validation cohorts to ensure consistency and stability across diverse patient populations.

### SHapley Additive exPlanations (SHAP)

#### Analysis for model interpretability

We applied SHAP [9] analysis to ensure interpretability of the ML model. The SHAP provides insights into how each feature contributes to the model's predictions by calculating the Shapley values, which represent the marginal contribution of each feature to the output. This technique explained the impact of each feature on the model's decision-making process for individual predictions and across the entire data set.

We generated the SHAP summary plot to visualise the importance of features and their corresponding influence on the model's output. The plot shows the distribution of SHA*P* values for each feature, indicating whether the feature had a positive or negative impact on the predicted outcome. By applying SHAP analysis, we aimed to enhance transparency in the model's decision-making, ensuring that the predictions were interpretable and actionable in a clinical context.

## RESULTS

### Clinical flowchart for HF and descriptive analyses

The inclusion criteria highlighted in the flowchart (**Figure 1**, Panel A) was based on a selection of ICD-10 coded diagnoses and performance of specific procedures offers a robust and clinically consistent criterion for identifying patients with a high probability of HF within the context of the *SIA-SUS*. For this purpose, we selected the ICD-10 codes I21 (acute myocardial infarction), I25 (chronic ischaemic heart disease), I42 (cardiomyopathy), and I48 (atrial fibrillation and flutter), as they represent conditions frequently associated with the development and progression of HF, and the inclusion of procedures such as BNP/NT-pro-BNP, echocardiography, cardiac catheterisation, ergospirometry, and serology for Chagas disease serving as an additional element to reinforce the identification of patients with a high probability of HF [5].

In contrast, to identify patients whose deaths were likely caused by underreported HF within the context of the *SIM-SUS* (**Figure 1**, Panel B) the inclusion criteria focused on the presence of cardiomyopathies, valvular heart diseases, myocardial infarction, and ischaemic heart diseases. These conditions were represented by the ICD-10 codes B57 (Chagas disease), I05-I08 (rheumatic heart diseases), I21-I25 (ischaemic heart diseases, including myocardial infarction), I34-I39 (non-rheumatic valve disorders), I42-I44 (cardiomyopathies), I48 (atrial fibrillation and flutter), I49 (other cardiac arrhythmias), and I51 (complications and ill-defined heart diseases). These codes were captured across lines A, B, C, D, and II of the *SIM-SUS* system to identify deaths potentially attributable to HF that may have been underreported officially.

Diagnosed HF patients and potential HF patients showed broadly similar mean ages, but potential HF cases were consistently slightly older (~ 61–63 years *vs*. 57–61 years across the period) (**Table 1**). The proportion of men was higher among potential HF patients (~ 54–55%) than among diagnosed HF patients (rising from 45% to 51% over time). This pattern suggests that older individuals, particularly men with cardiovascular comorbidities, may be more likely to follow care trajectories consistent with HF without ever receiving an explicit I50 code in *SIA-SUS*. The total number of potential HF patients also remained substantial in all years (~ 42 000–51 000 per year), reinforcing the hypothesis that a non-trivial fraction of HF-like cases may be underreported in ambulatory data.

**Table 1:** Description of the population of confirmed patients and potential cases of HF

	Age at diagnosis in years, x̄ (SD)	Male, n (%)	Patients, n
**Diagnosed**			
2018	57.25 (21.35)	32 674.00 (45.19)	72 309
2019	57.83 (21.33)	35 637.00 (46.02)	77 445
2020	59.86 (19.75)	30 663.00 (47.98)	63 905
2021	60.26 (19.16)	44 431.00 (48.91)	90 842
2022	61.15 (18.18)	62 291.00 (50.58)	123 155
**Potential HF**			
2018	61.02 (14.55)	26 462.00 (53.74)	49 242
2019	61.94 (14.06)	28 810.00 (54.71)	52 659
2020	62.45 (13.41)	23 556.00 (55.29)	42 603
2021	62.48 (13.96)	28 139.00 (55.19)	50 986
2022	62.66 (14.49)	28 136.00 (54.79)	51 351

HF – heart failure, SD – standard deviation, x̄ – mean

Deaths among patients with a confirmed HF diagnosis occurred at a higher mean age (~ 75 years), with a nearly equal sex distribution (**Table 2**). In contrast, potential HF-related deaths occurred at a slightly lower mean age (around 71 years) and showed a higher proportion of men (~ 56–57%). The absolute number of potential HF-related deaths was also markedly higher than confirmed HF deaths in every year, ranging from about 188 000 to 214 000 deaths per year *vs.* 108 000 to 132 000 confirmed HF deaths. These differences are consistent with the notion that a large number of deaths in individuals with HF-compatible conditions may be coded under other cardiovascular causes (*e.g.* ischaemic heart disease or cardiomyopathies), thereby masking the true mortality burden attributable to HF.

**Table 2:** Description of confirmed patient deaths and potential HF-related deaths

	Age on day of death in years, x̄ (SD)	Male, n (%)	Number of deaths
**Diagnosed**			
2018	74.68 (17.42)	53 239.00 (49.10)	108 430
2019	74.88 (17.14)	53 935.00 (49.14)	109 758
2020	74.79 (15.98)	58 719.00 (49.83)	117 849
2021	74.88 (16.02)	65 535.00 (49.65)	131 981
2022	75.36 (16.41)	64 342.00 (48.96)	131 411
**Potential HF**			
2018	70.75 (21.57)	105 724.00 (56.28)	187 856
2019	70.97 (21.16)	107 688.00 (56.20)	191 612
2020	71.30 (19.20)	110 659.00 (57.01)	194 111
2021	71.12 (19.07)	120 797.00 (56.55)	213 606
2022	71.47 (19.52)	113 179.00 (56.19)	201 418

HF – heart failure, SD – standard deviation, x̄ – mean

This analysis, which shows the absolute number of deaths related to potential cases of HF, stratified by ‘proxy’ ICDs, specifically cardiomyopathies and heart attacks and ischaemia, which are conditions that make up potential cases of HF (Figure S3 in the **Online Supplementary Document**), indicates that most deaths associated with potential HF cases are attributed to cases of heart attacks and ischemia, representing approximately 180 000 to 200 000 cases, which is equivalent to approximately 98% of potential HF cases. There is also a slight increase in the number of deaths from the year 2021 onwards. This concentration of potential HF-related deaths in patients coded with acute myocardial infarction and ischaemic heart disease supports the idea that HF frequently coexists with, or develops as a consequence of, ischaemic events, but is not always recorded as the primary or underlying cause of death in administrative data.

We conducted a deterministic sensitivity analysis to understand the impact of the underestimation of potential HF patients on the number of cases in Brazil and estimate the percentage of underestimation of ambulatory patients, as well as the percentage of underestimation of deaths among these patients during the five-year period (Figure S4 in the **Online Supplementary Document**). When there was a 20% underestimation in the number of potential HF cases, the underestimation rate of cases diagnosed with HF was 12%. For example, when there was an underestimation of 80% in potential HF cases, the underestimation of the number of cases diagnosed with HF reached 35%. We observed variations from 2021 onwards, and similar results can be seen in relation to deaths due to HF. When there was an underestimation of 40% in potential cases of death due to HF, there was an average underestimation of 41% in the number of deaths diagnosed as HF. In the scenario in which all potential patient deaths were HF cases, ~ 63% of the number of deaths diagnosed as HF were also underestimated. These scenario-based results indicate that, even under very conservative assumptions (*e.g.* only 20% of potential HF cases or deaths being true HF cases), the underestimation of ambulatory HF cases and HF-related deaths would already be epidemiologically meaningful (on the order of 10–15%). Under more liberal, yet still plausible assumptions (40–60%), underestimation rates rise to approximately 30–40%. We emphasise that the 0% and 100% scenarios represent theoretical bounds rather than realistic estimates; our interpretation focuses on these intermediate ranges, which align more closely with prior literature on HF underdiagnosis and misclassification.

### ML Model for HF patient identification

**Figure 2:**
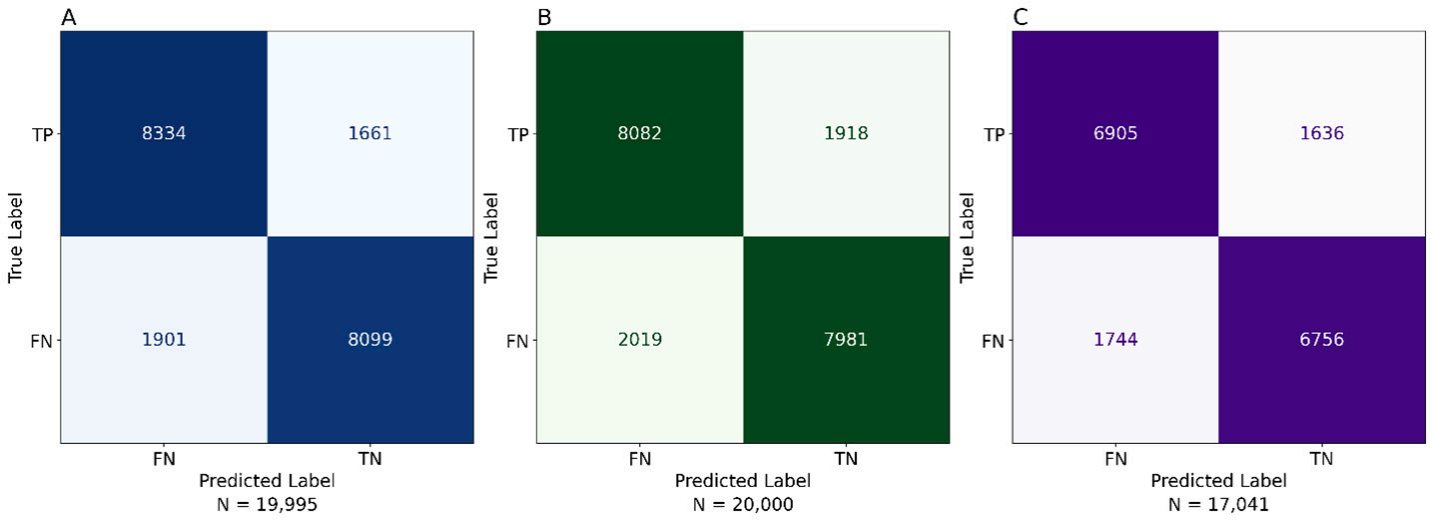
Confusion matrix analysis for training and validation data sets.

**Figure 3:**
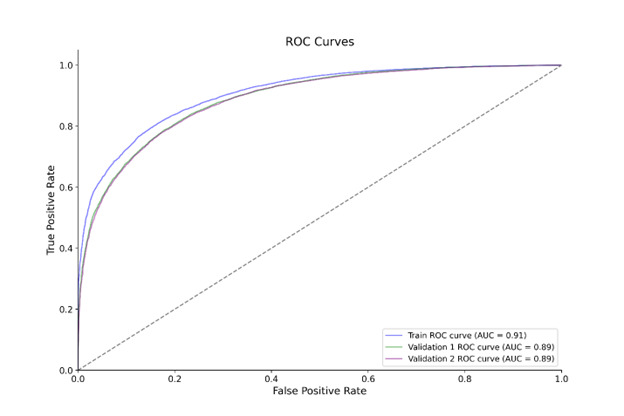
ROC curves for training set and validation sets.

We developed a ML model based on the analysed variables to determine a proxy for identifying patients with HF. The CatBoost model achieved the best performance using default parameters, with an accuracy of 0.7809, AUCROC of 0.8736, and recall of 0.7762 (**Table 3**). Consequently, we selected this model for further improvement. We performed a ‘randomsearch’ algorithm and ‘skopt’ optimisation, followed by ‘grid search’ for fine-tuning. We used the following parameters in this model: learning_rate (0.083), l2_leaf_reg (25), iterations (900), depth (8), border_count (30) (Table S2 in the **Online Supplementary Document**). We presented evaluation metrics, including AUC, accuracy, F1-Score, recall, and Brier score, for the training set and two validation sets. The confusion matrices for the training set and validation sets (**Figure 2**) shows the distribution of true and false predictions. The training set indicated a balanced rate, while the validation sets provide insights into model performance on unseen data. The model demonstrated solid performance with an accuracy of 82.2% on the training dataset (Table S3 in the **Online Supplementary Document**), maintaining similar accuracy across validation sets (80.3% for validation set 1 and 80.2% for validation set 2). The ROC curves (**Figure 3**) demonstrate the model’s performance across data sets, with a training AUC of 0.91 and AUC values of 0.89 for validation sets. These results indicate that the model generalised well, maintaining robust performance across all data sets.

**Table 3:** Summary of accuracy, AUC ROC, and recall for various ML algorithms

ML Algorithms	Accuracy	AUC ROC	Recall
Random Forest	0.7632	0.8414	0.7607
GradientBoosting	0.7642	0.8596	0.7632
SVM	0.7449	0.8332	0.6269
CatBoost	0.7809	0.8736	0.7762
Logistic Regression	0.793169	0.793169	0.702857
XGBoost	0.7824	0.8717	0.7752
AdaBoost	0.7684	0.8585	0.7572

AUC – Area Under the Curve, ML – machine learning, ROC – Receiver Operating Characteristic, SVM – Support Vector Machine

When we applied the model trained on the balanced data set to the low-prevalence evaluation cohort (175 HF cases among 8716 individuals; prevalence ~ 2%), it maintained strong discriminative performance (Table S4 in the **Online Supplementary Document**). CatBoost achieved an AUC of 0.837 and preserved high sensitivity (0.823), indicating that the model remained capable of identifying most HF cases even in a real-world imbalanced context. As expected in rare-outcome scenarios, overall accuracy (66.4%) was dominated by the majority class, and the F1-score declined markedly (0.089), reflecting the inherently low positive predictive value associated with very low event prevalence. Calibration remained acceptable, with a Brier score of 0.189.

When compared with logistic regression under the same conditions, CatBoost demonstrated superior discriminative ability (AUC of 0.837 *vs.* 0.793), higher sensitivity (82% *vs.* 70%), and better calibration (Brier score of 0.189 *vs.* 0.211) (Table S4 in the **Online Supplementary Document**). Although logistic regression produced higher accuracy, this was largely attributable to predicting a greater number of negative cases and did not translate into improved detection of HF. Precision and F1-score were low for both models, which is expected in data sets with extreme class imbalance and low true event counts.

Overall, CatBoost provided a more clinically meaningful balance between discrimination and sensitivity in identifying HF cases within a population reflecting real-world HF prevalence. In practical terms, this means that, if used as a screening or risk-stratification tool at the population level, the CatBoost model would be able to flag the majority of true HF cases for further clinical evaluation, accepting a larger number of false positives as an inherent trade-off in low-prevalence settings.

The CatBoost model demonstrated globally good calibration across all data sets (Figures S5 and S6 and Table S5 in the **Online Supplementary Document**). Brier scores ranged from 0.124 in the training set to 0.136 in the validation set, indicating excellent overall agreement between predicted probabilities and observed outcomes. Expected calibration error was very low (0.03 in the training set and 0.01–0.02 in the validation sets), and the maximum calibration error did not exceed 8 percentage points in any probability bin. Calibration intercepts were close to zero (from –0.03 to +0.03), and calibration slopes were near 1.0 in the validation cohorts (1.10 and 1.07), suggesting only mild under confidence of the predictions.

Although the Hosmer-Lemeshow test was statistically significant in all data sets (*P* < 0.01), this likely reflects the large sample size and the known sensitivity of this test to minor deviations from perfect calibration. Taken together with the low Brier scores, small ECE and MCE, and the visual inspection of calibration plots, these findings support that the model is well calibrated in a clinically meaningful sense, with only small deviations that are unlikely to affect decision-making.

To better understand the contribution of each feature in the model, we conducted a SHAP analysis (Figure S7 in the **Online Supplementary Document**) that highlights the features that most impact the model’s prediction, including transthoracic echocardiogram, chronic kidney disease, age in years, race, and lipoprotein metabolism disorders. Interestingly, some of the identified features (Figure S7 in the **Online Supplementary Document**), such as echocardiogram and acute myocardial infarction (I21), align with those outlined in the clinical proxy HF identification (**Figure 1**), indicating strong concordance between the model’s key features and the established clinical criteria. This convergence between model-derived feature importance and the expert-designed proxy criteria increases confidence that the model is capturing clinically meaningful HF-related patterns rather than artefacts of coding or noise.

The clinical proxy was fundamental to validating the model’s performance. We used the cohort of patients classified as potential HF cases by the clinical proxy (**Figure 1**). This cohort, totalling 76 757 patients, comprised individuals recorded in the *DATASUS* database between 2018 and 2021, with available data on race, weight, and height. Among these, 71 242 were also identified by our ML model as having HF. This substantial overlap, where 92.81% of the individuals identified by the proxy were similarly classified by the model, highlights the strong internal consistency between the proxy and the ML model and suggests that the model is largely learning patterns consistent with established clinical reasoning, while also integrating more complex nonlinear combinations of features.

## DISCUSSION

To the best of our knowledge, this is the first study to use administrative public database of Brazil to jointly explore the potential misdiagnosis and underdiagnoses of HF and its associated mortality burden. It is well known that HF is a common syndrome that represents the end stage of several heart diseases. Decompensation is the main cause of hospitalisations in developed countries and the third general cause of hospitalisation in Brazil [4]. In this study, we combined a clinically grounded proxy (**Figure 1**) and a ML predictive model to approximate the ‘hidden’ ambulatory and mortality burden of HF in *DATASUS* and to identify individuals at increased risk of HF before a formal I50 code appears.

Recognising that many cases of hospitalisations due to HF are underreported because they are associated with other pathologies, especially in cases where HF presents with mild symptoms, we created a clinically grounded flowchart to identify potential HF patients. The inclusion criteria outlined in the flowchart (**Figure 1**) strategically combine selected ICD-10 diagnoses and key investigative procedures, providing a structured framework for identifying patients with a high probability of HF within the *SIA-SUS* context. This approach is grounded in strong clinical associations between the selected conditions and progression towards HF, particularly when these conditions are investigated through core diagnostic exams.

For example, chronic ischaemic heart disease is the major underlying pathogenic factor for HF, increasing the risk of HF 8-fold, and has been established as an important prognostic factor for the condition [10]. Patients with this condition are frequently monitored with BNP/NT-pro-BNP testing, well stablished biomarker of HF [11]. The routine use of these tests in this patient population increases the likelihood of identifying those with underlying HF that may not yet be clinically evident.

Demographic data showed a similar number of HF cases between men and women, with a slight predominance of men in the cohort of potential HF. We also observed an increase in the number of deaths over the study period. This phenomenon could be attributed to ageing, which is a significant factor in the development and progression of HF [12], and to improved recognition and coding of HF over time [13]. Importantly, the COVID-19 pandemic contributed to lower reported numbers of many conditions worldwide during 2020, including HF. The decrease in hospitalisations for non-COVID-19 reasons appears to be linked to hospital overcrowding during periods of COVID-19 surges, coupled with reduced demand for healthcare from individuals concerned about contracting the virus [14].

A systematic review analysed the burden of HF in Latin American countries in 2016 based on 143 studies, 64% of which were conducted in Brazil, and reported an HF incidence of 199 cases per 100 000 persons-years [15]. The mean percentage of men was 61.07% (standard deviation (SD) = 11.48%) with a mean age of 60.34 (SD = 8.98) years [15]. We found that the age at diagnosis of HF was similar between confirmed HF and potential HF patients (around 59 years and 61 years, respectively), and the proportion of men was slightly higher among potential HF patients. These similarities support the clinical plausibility of our proxy and suggest that it aligns a population with demographic characteristics consistent with those reported in previous Brazilian and Latin American studies.

We rigorously selected the inclusion criteria based on the Brazilian Guidelines for HF [5]. The presence of diseases well established as conferring high risk for HF, combined with HF-related procedures, likely indicates an ongoing investigation or evaluation for HF. This gives us reason to hypothesise that many of these patients have an increased probability of underlying HF. To explore how these potential cases might affect estimates of HF burden, we performed a deterministic sensitivity analysis, explicitly designed to reflect uncertainty around the true proportion of potential cases that genuinely have HF. By varying the assumed proportion of potential cases that correspond to true HF (from 0% to 100%), we obtained a range of plausible underestimation rates rather than a single point estimate (Figure S4 in the **Online Supplementary Document**).

Even under conservative assumptions that only 20% of potential ambulatory HF cases correspond to true HF, the analysis suggests an underestimation of diagnosed ambulatory HF cases of approximately 12%. Under more liberal, yet still plausible assumptions (*e.g.* 40–60% of potential cases being true HF), underestimation rates rise to approximately 30–40%. Our findings are therefore consistent with the hypothesis that a substantial fraction of HF in Brazil may be underdiagnosed or misclassified, particularly among patients with competing comorbidities or mild, non-specific symptoms. However, our estimates are derived from proxy-based definitions and predictive modelling, rather than direct clinical adjudication. As such, they should be interpreted as upper-bound approximations of the potential burden of underrecognised HF, not as definitive measurements of untreated disease. Future studies incorporating chart review, echocardiographic data, and biomarker confirmation will be necessary to validate the true proportion of misdiagnosis and under-treatment in this population.

Our interpretation aligns with prior work showing that HF is particularly prone to misdiagnosis when patients present with mild, non-specific, or overlapping symptoms. In the systematic review by Wong et al [16], misdiagnosis ranged from 16.1% among patients discharged from the hospital with a diagnosis of HF to 68.5% among primary care referrals for suspected HF who did not have left ventricular dysfunction, valvular disease, or atrial fibrillation. Common contributors to diagnostic error included dyspnoea and fatigue due to alternative conditions, limited use of natriuretic peptides, and underutilisation of echocardiography, especially in primary care. Similar patterns have been described in patients with chronic obstructive pulmonary disease (COPD) and type 2 diabetes [17,18], in whom chronic HF is often overlooked or mislabelled despite a high underlying risk. Our findings support our hypothesis that mild or nonspecific symptomatology and multimorbidity (*e.g.* COPD, diabetes, CKD) contribute substantially to the under-recognition and misclassification of HF in Brazilian administrative data.

Our results also need to be interpreted in the context of Brazilian coding practices and structural heterogeneity within SUS. Because *SIA-SUS* requires an ICD-10 code to authorise most procedures, clinicians may use codes that reflect diagnostic suspicion, comorbidities, or symptoms, rather than confirmed HF. In principle, this raises the possibility that our proxy and ML model could be overfitting to local coding behaviour, that is, learning patterns of documentation and reimbursement rather than the underlying clinical syndrome. This concern is partially mitigated by the strong overlap between model features and guideline-based clinical criteria, as well as by the biological plausibility of the top predictors (*e.g.* CKD, ischaemic heart disease, age, echocardiography) [5]. Still, we cannot exclude the possibility that regional differences in coding habits, resource availability, and medical training influence both the proxy and the model’s performance. For example, regions with fewer cardiologists or limited access to echocardiography may underutilise HF-related procedures, leading to systematic under-capture of true HF and potential geographic variability in model calibration.

Prior studies highlighted that HF misdiagnosis is often driven by overlapping symptomatology, variable access to diagnostics, and inconsistent use of natriuretic peptides or imaging [16,17]. Overall, this body of evidence supports the use of a proxy-based and sensitivity-driven approach to estimate the hidden burden of HF in settings where diagnostic uncertainty and incomplete coding limit direct epidemiological measurement, as in *DATASUS*. Our approach complements this literature by demonstrating that large-scale administrative data can be used to approximate the hidden burden of HF, but does not fully resolve the underlying diagnostic uncertainty. Moreover, our study does not stratify model performance by region, level of care (primary *vs.* specialised), or socioeconomic indicators, which are known to shape both healthcare access and data quality in Brazil. Future work should explicitly examine how these structural factors modulate both the likelihood of receiving an I50 code and the reliability of proxy-based phenotyping, ideally by linking *DATASUS* records to more granular clinical registries or hospital information systems. This is an important limitation of our analysis, and future studies should undertake region-specific evaluations and link administrative data to richer clinical information systems to better characterise and correct for these sources of bias. Several factors inherent in HF are frequently misdiagnosed due to the presentation and clinical assessment [19], delaying proper treatment. Symptoms such as shortness of breath, fatigue, and oedema can overlap with those of other cardiovascular and respiratory conditions, leading to diagnostic ambiguity [20]. For example, COPD can present with similar symptoms to those of HF [14]. Moreover, the clinical manifestations of HF may vary widely among patients, making it challenging to recognise consistently across different individuals. Additionally, healthcare providers may encounter difficulties in interpreting diagnostic tests accurately, especially in cases where imaging and biomarkers yield inconclusive results or where comorbid conditions complicate the diagnostic process [21,22]. Ultimately, the misdiagnosis of HF underscores the importance of enhanced clinical awareness, comprehensive evaluation strategies, and continued medical education to improve diagnostic accuracy and optimise patient outcomes. We designed the deterministic sensitivity analysis explicitly to reflect uncertainty around the true proportion of potential cases that genuinely have HF. By varying the assumed proportion of potential cases that correspond to true HF (from 0% to 100%), we provide a range of plausible underestimation rates rather than a single point estimate. Even under conservative assumptions (*e.g.* 20% of potential ambulatory HF cases being true HF), the analysis suggests non-trivial levels of underreporting. Nonetheless, these scenarios remain hypothetical, and the study does not directly confirm clinical diagnoses for individuals classified as ‘potential HF’ by the proxy or the model.

To better understand this scenario and to enable early diagnoses, we developed a ML predictive model using the CatBoost algorithm. The idea was to identify patients in the *SIA-SUS* system before they were diagnosed with ICD-10 code I50 (HF). Importantly, the *DATASUS* system often requires clinicians to assign ICD-10 codes even when a diagnosis is still under investigation, which may lead to the early appearance of disease codes. By restricting predictors to ≥12 months before the first I50 code and excluding all data from the diagnostic workup period, we minimised the risk of data leakage. Therefore, the model’s performance reflects its ability to identify individuals at increased risk prior to clinical suspicion or coding, supporting its potential use for early detection and risk stratification. ML models could detect patterns in the *SIA-SUS* system, acting as an ally to physicians in diagnosing HF. By facilitating earlier diagnoses, this model may improve patient outcomes and reduce the risk of misdiagnosis. Furthermore, this approach could help lower healthcare costs in Brazil by minimising unnecessary treatments and redirecting patients to the correct treatment pathways, ultimately reducing the risk of death from HF. To our knowledge, this is the first work to develop a ML model for use in the Brazilian health system, *DATASUS*, to predict HF. The model achieved an AUCROC of 0.91, with an accuracy of 0.89 and a recall of 0.89 for the training set. The excellent calibration of our model within this data set further supports its potential for use in risk stratification, although external clinical validation is still required before routine implementation. Brier scores ranged from 0.124 to 0.136 across data sets, indicating strong agreement between predicted probabilities and observed outcomes. Calibration slopes near 1.0 (1.07–1.10) in the validation cohorts suggest only mild underconfidence, which is clinically preferable to overconfident predictions that could lead to inappropriate certainty in diagnosis. Although the Hosmer-Lemeshow test was statistically significant (*P* < 0.01), this likely reflects the test's sensitivity to minor deviations in large samples rather than clinically meaningful miscalibration, as evidenced by low expected calibration errors (0.01–0.03) and maximum calibration errors below 8 percentage points. These calibration properties ensure that the model's probability estimates can be reliably used to stratify patients by risk, facilitating targeted screening and resource allocation within the Brazilian public health system. Other HF models have also been developed with different types of predictor features, with their AUC values varying in specificity among 0.7 to 0.94 [23].

A crucial question for any predictive model trained on a balanced sample is whether its performance generalises real-world scenario low prevalence. In the validation cohort reflecting a realistic population prevalence of HF (~ 2%), CatBoost demonstrated superior performance compared with logistic regression, particularly in metrics most relevant for early detection. Although both models showed the expected decline in precision and F1-score due to the rarity of HF, CatBoost maintained higher discrimination (AUC 0.837 *vs.* 0.793) and substantially greater sensitivity (0.823 *vs.* 0.703), while also exhibiting better probability calibration (Brier score 0.189 *vs.* 0.211). Logistic regression, although achieving higher accuracy, did so largely by favouring the negative class, which is of limited clinical value in a screening context where missed HF cases are particularly harmful. The improved sensitivity and calibration of CatBoost suggest that more complex, nonlinear models may capture subtle interactions between comorbidities, diagnostic procedures, and demographic variables that traditional linear models fail to. This is especially relevant in a database such as *DATASUS*, characterised by noisy, heterogeneous, and often incomplete information.

Our findings compare favourably with previous international studies that used large-scale EHR data to predict HF. For example, models developed using the Geisinger health system achieved AUCs of 0.74–0.77, while other HF prediction models in the literature report AUCs of 0.70–0.94 depending on the population, predictors, and endpoints considered [22]. The AUC of 0.91 observed in our study, combined with strong calibration, is particularly notable given that *SIA-SUS* lacks laboratory and imaging results (only procedure requests and ICD justifications are available), and that coding practices in the Brazilian public system often reflect diagnostic suspicion rather than confirmed disease. In this challenging data environment, the model’s performance suggests that it can extract meaningful prognostic signals from patterns of consultations, procedures, and comorbid ICD codes, rather than relying on high-resolution clinical measurements.

Importantly, the interpretability analysis using SHA*P* values reinforces the clinical plausibility of the model. The most influential predictors, such as transthoracic echocardiography, chronic kidney disease, age, race, and lipoprotein metabolism disorders, are not only consistent with established HF pathophysiology but also overlap substantially with the criteria used in our clinically derived proxy. Procedures such as echocardiography, cardiac catheterisation, and electrocardiography, along with diagnoses such as ischaemic heart disease and acute myocardial infarction, emerged as key features influencing HF prediction, in line with Brazilian HF Guidelines [5].

Furthermore, CKD, the second most important feature according to SHAP analysis (Figure S7 in the **Online Supplementary Document**) is a well-established risk factor for HF. Renal dysfunction and damage significantly increase the prevalence of HF, particularly in cases of HF with preserved ejection fraction among older adults [24]. In addition to lipoprotein metabolism disorders, which can increase the risk of cardiovascular diseases [5,25], including coronary artery disease, peripheral artery disease, stroke, and HF, by promoting atherosclerosis and its complications [25]. The strong agreement between the model’s feature importance profile and the expert-designed clinical flowchart is further underscored by the fact that 92.8% of patients classified as potential HF by the clinical proxy were also identified as HF by the ML model. This high concordance suggests that the model is essentially ‘learning’ the same clinical logic, while adding the ability to integrate complex, nonlinear combinations of predictors that are difficult for clinicians to discern in routine practice. From a health system perspective, these results have relevant practical implications. First, the model could be embedded within *DATASUS* or regional analytic platforms to periodically scan ambulatory records and generate lists of patients at high risk for HF, before a formal I50 code appears. These individuals could be prioritised for confirmatory evaluation (*e.g.* cardiology consultation, echocardiography, natriuretic peptide measurement) or enrolled in monitoring programs, especially in regions with limited access to specialists. Second, the good calibration of the predicted probabilities allows health managers to define risk thresholds tailored to available resources, for example, focusing on the top 5–10% of predicted risk to maximise yield while maintaining operational feasibility. Third, by facilitating earlier diagnosis and more appropriate care pathways, this approach has the potential to reduce avoidable decompensations, hospitalisations, and HF-related deaths, with positive implications both for patient outcomes and for the financial sustainability of SUS.

Finally, beyond its immediate predictive performance, this study demonstrates the feasibility and value of applying advanced ML methods to large administrative health databases in low- and middle-income settings. To our knowledge, this is the first work to develop and validate an ML model specifically tailored to *DATASUS* for HF prediction. In a context where underreporting and misclassification of HF are substantial, the integration of such models with clinically grounded proxies may help to uncover the ‘hidden burden’ of HF, inform more accurate estimates of disease prevalence and mortality, and guide public policies aimed at improving early detection, optimising resource allocation, and reducing inequities in cardiovascular care across Brazil.

From a policy perspective, our findings suggest several complementary roles for the proposed model within the Brazilian health system. Given its high sensitivity and good negative predictive value, particularly in low-prevalence settings, the model is best conceptualised as a screening and risk-stratification tool, rather than a stand-alone diagnostic test. At the population level, it could be integrated into analytic pipelines within *DATASUS* or state-level health departments to periodically flag individuals at high predicted risk of HF who have not yet received an I50 code. These patients could then be prioritised for targeted clinical review, structured follow-up, or referral for confirmatory evaluation (*e.g.* cardiology consultation, echocardiography, natriuretic peptide testing), depending on local resource constraints.

In routine practice, model outputs could be presented to clinicians as risk scores or alert lists embedded within existing information systems, clearly labelled as probabilistic risk rather than definitive diagnoses. For example, primary care teams might receive periodic reports identifying patients in their catchment area who fall into the top decile of predicted HF risk, prompting chart review and, when appropriate, diagnostic workup. At the health system level, the model could also serve as a retrospective audit mechanism, helping managers identify patterns of undercoding or potential gaps in HF detection across regions, facilities, or demographic groups. Any such implementation would need to be incremental and accompanied by training for clinicians and managers on how to interpret and act on risk predictions, as well as by continuous monitoring of calibration and fairness across subpopulations. At this stage, the model should therefore be viewed as a decision-support and surveillance tool to augment, rather than replace, clinical judgment and guideline-based evaluation.

The ethical implications of false positives are particularly salient in predictive modelling for chronic diseases such as HF. In our low-prevalence evaluation, the model maintained high sensitivity but, as expected, had a relatively low positive predictive value, meaning that many patients flagged as ‘high risk’ will not ultimately have HF. If used uncritically, this could lead to unnecessary investigations, patient anxiety, and potential overtreatment, as well as misallocation of already constrained diagnostic resources. To mitigate these risks, model outputs should not trigger automatic therapeutic decisions; rather, they should be used to prioritise careful clinical assessment, guided by established HF guidelines and incorporating patient preferences and shared decision-making. These safeguards are essential to ensure that the model augments, rather than undermines, equitable and patient-centred care.

From a health-system standpoint, an excessive focus on maximising sensitivity without considering downstream consequences could exacerbate inequities, especially if additional diagnostic capacity is not distributed evenly across regions. Implementations should therefore be accompanied by explicit thresholds and protocols that balance sensitivity with feasibility, for example, limiting proactive workup to the highest-risk strata, or combining model predictions with simple clinical screening tools (*e.g.* symptom checklists, vital signs) before ordering more expensive tests. Moreover, continuous evaluation of real-world performance, monitoring rates of confirmed HF among flagged individuals, resource utilisation, and patient-reported outcomes, will be essential to ensure that the model improves care without generating disproportionate harms. These considerations align with broader ethical frameworks for AI in healthcare, which emphasise transparency, proportionality of interventions, and ongoing evaluation of unintended consequences when deploying predictive models in routine care.

Shifting focus to mortality (**Table 2**) and the sensitivity analysis (Figures S3 and S4 in the **Online Supplementary Document**) showed that if an underestimation of 40% in potential cases of death due to HF, an average underestimation was 41% in cases of death diagnosed as HF. Additionally, we represented the number of deaths in subgroups of diseases such as heart attack and ischaemia and cardiomyopathies, showing that there is a predominance in the first subgroup over the second. Almost all deaths flagged as potential HF are from patients with heart attack and ischemia as their main cause. It is well known that HF often accompanies other cardiovascular conditions, compounding the risk of adverse outcomes. Early diagnosis, comprehensive management strategies, adherence to treatment plans, and lifestyle modifications play pivotal roles in reducing mortality rates and improving survival outcomes among individuals living with HF.

Our data point to an overall increase in HF-related ambulatory patients and mortality and potential HF cases over five years, with notable fluctuations and significant variations in specific years. Furthermore, the underestimation of potential HF cases potentially impacted the underestimation of diagnosed cases and deaths from HF, highlighting the importance of an accurate assessment of these data for the effective management of this health condition.

By integrating these mortality insights with our ML model's robust predictive capabilities, which demonstrated high alignment with clinical criteria and highlighted key risk factors such as CKD, age, and lipoprotein metabolism disorders, we underscore the potential of using ML as a tool to enhance early detection and monitoring of HF patients. The substantial overlap between the model's features and clinical proxies not only supports the internal validity of the model’s predictions but also reinforces its potential application in clinical practice to address the challenges of HF diagnosis and management. This comprehensive approach, combining ML insights with an understanding of mortality trends, while recognising that our estimates of underestimation and mortality remain model-based and require confirmation in clinical and prospective studies.

### Limitations

We found important limitations inherent to using administrative data and proxy-based phenotyping. First, *DATASUS*/*SIA-SUS* captures procedures and ICD-10 justifications for reimbursement, but lacks clinical results (*e.g.* echocardiography findings, natriuretic peptide values) and does not provide adjudicated diagnoses; ICD codes may reflect diagnostic suspicion rather than confirmed disease, and reporting quality may vary across regions and levels of care within SUS. Second, our ‘potential HF’ definition is a clinically grounded proxy that may misclassify patients (both false positives and false negatives), particularly in multimorbid individuals where symptoms and investigations overlap across conditions; therefore, the deterministic underestimation estimates should be interpreted as scenario-based approximations rather than definitive measurements. Third, although we applied strict temporal windowing to reduce leakage, the model may still learn patterns of documentation and care pathways specific to claims data, raising the possibility of overfitting to local coding behaviour; external validation against richer clinical data sets (chart review, imaging, biomarkers) remains necessary. Finally, feasibility and impact were not tested prospectively, and the study did not evaluate regional calibration or performance differences by healthcare access, which could influence both coding and model outputs.

## CONCLUSIONS

Our results highlight the importance of accurate diagnosis and the need for a comprehensive approach to identifying potential cases of HF to improve the recording and management of this condition in Brazil. The underestimation of HF cases suggested by our proxy and sensitivity analyses may have meaningful implications for public health and clinical management of HF, emphasising the need for strategies to promote early detection and more complete case recording.

Within this context, the ML model we developed, specifically tailored to *DATASUS*, showed promising discriminative performance and clinically meaningful calibration, and its most influential predictors were closely aligned with clinically established HF pathophysiology and guideline-based criteria. Rather than providing definitive diagnoses, the model should be viewed as a decision-support and surveillance tool that can help flag individuals at increased risk of HF before a formal code identification, thereby supporting clinicians and health managers in prioritising these patients for further evaluation.

While the use of prediction tools in public health initiatives such as *DATASUS* has the potential to reduce gaps in HF diagnosis, improve the tracking and management of potential cases, and contribute to more efficient use of SUS resources, our findings are based on proxy definitions and administrative data and do not replace clinical adjudication. Future work should therefore focus on validating these approaches against richer clinical data sets, assessing their real-world impact on patient outcomes and resource allocation, and developing implementation frameworks that address ethical, operational, and equity considerations within the Brazilian health system.

## Additional material


Online Supplementary Document
