# Supplementary material for: Unveiling the gap in heart failure: a Brazilian Unified Health System study
Source: J Glob Health. 2026 May 15;16:04097. doi: 10.7189/jogh.16.04097 (PMC13178052; doi:10.7189/jogh.16.04097)
Supplement: Online Supplementary Document [file jogh-16-04097-s001.pdf]

Supplement to: Volpi e Silva N, Vicentini D, Vitorasso RL, Nogueira-Pileggi V, Bilevicius E, Batista VC, Picoli RM. Unveiling the gap in heart failure: a Brazilian Unified Health System study. J Glob Health. 2026;16:04097.

Figure S1. ROC Curves using Cross-validation = 5 to Training set and Validation Sets.

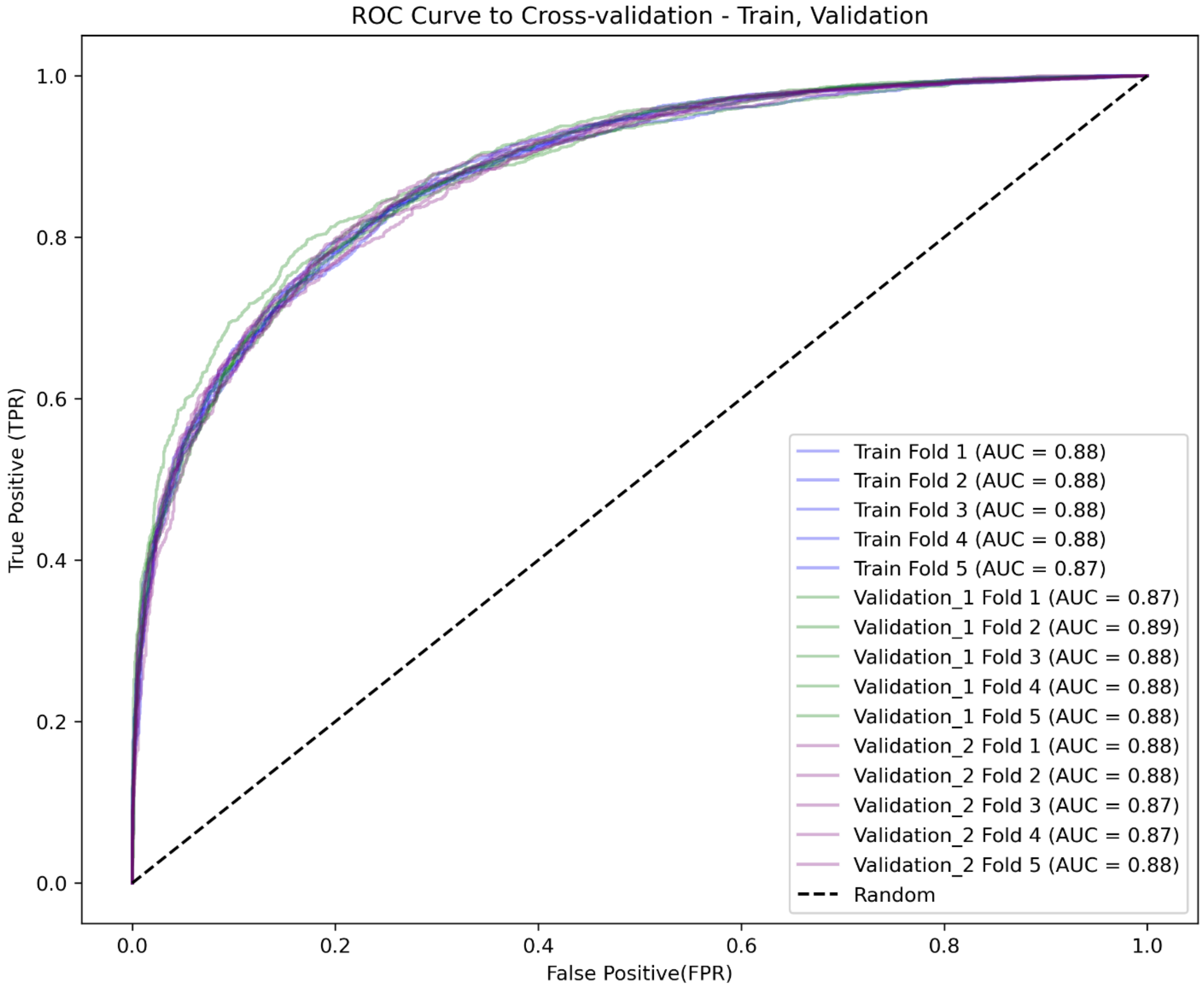

Figure S2. Distribution of predicted probabilities for HF and non-HF patients in each dataset.

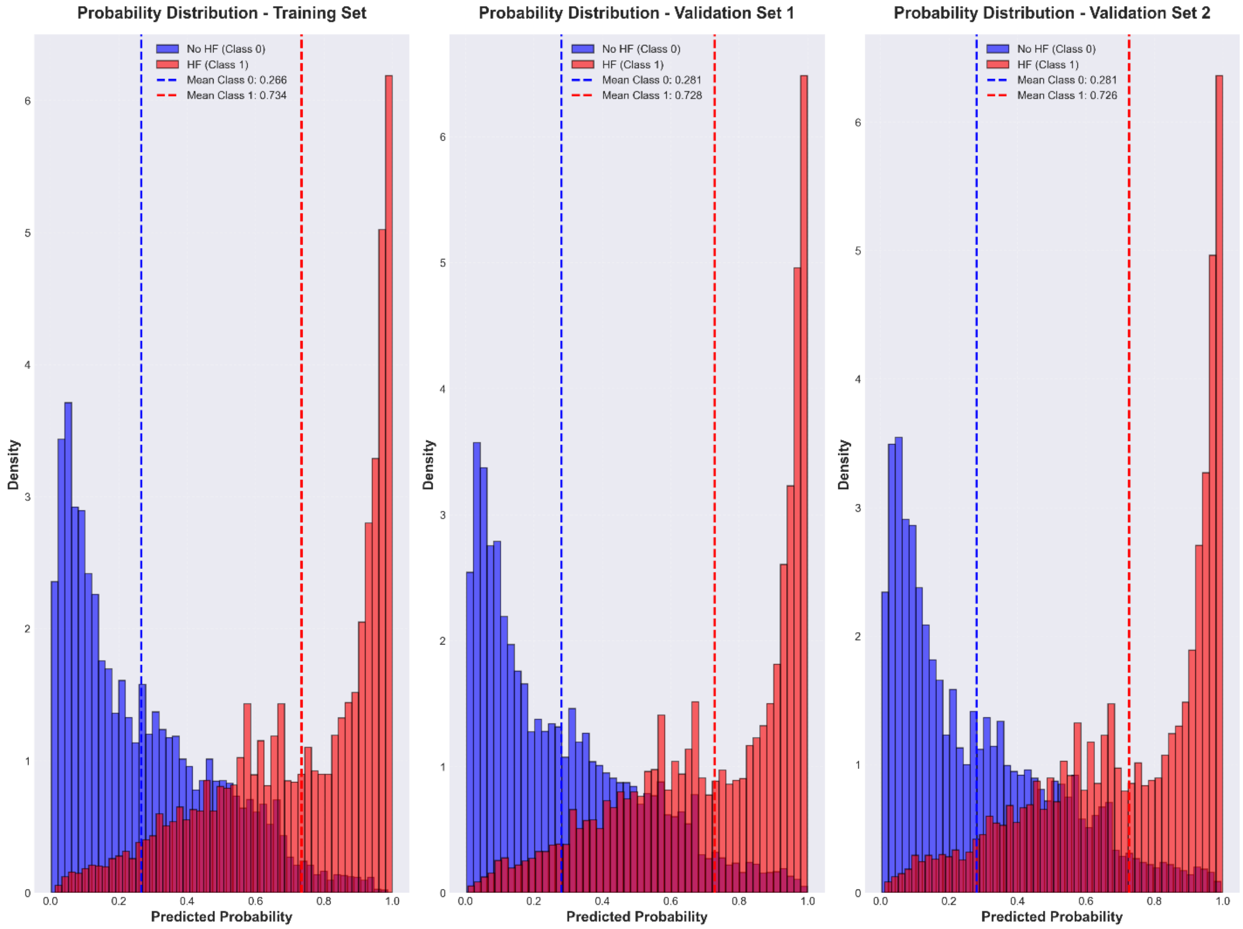

Figure S3. Total number of deaths of patients classified as potential patients with HF divided into the following groups: 1) Cardiomyopathy, valvular heart disease and valvular heart disease; and 2) Ischemia and heart attacks.

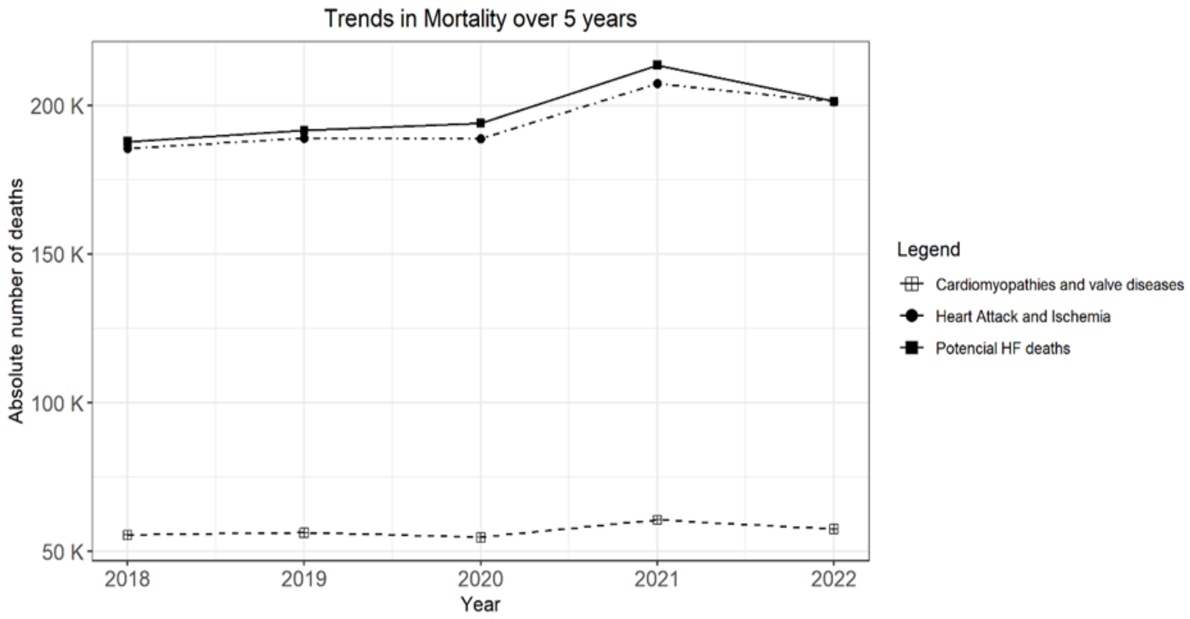

Figure S4. Sensitivity analysis of the rate of underestimation of A) ambulatorial diagnosis and B) deaths related to HF over the time interval studied. The sensitivity analysis will be carried out considering values from 0% to 100% (0; 20; 40; 60; 80; 100%) as the proportion of potential patients/deaths as actual patients/deaths related to HF. Example, to calculate the underestimation rate of deaths at 20%: (potential number of deaths related to HF * 20%) / (potential number of deaths related to HF * 20% + number of deaths with I50 registration).

A

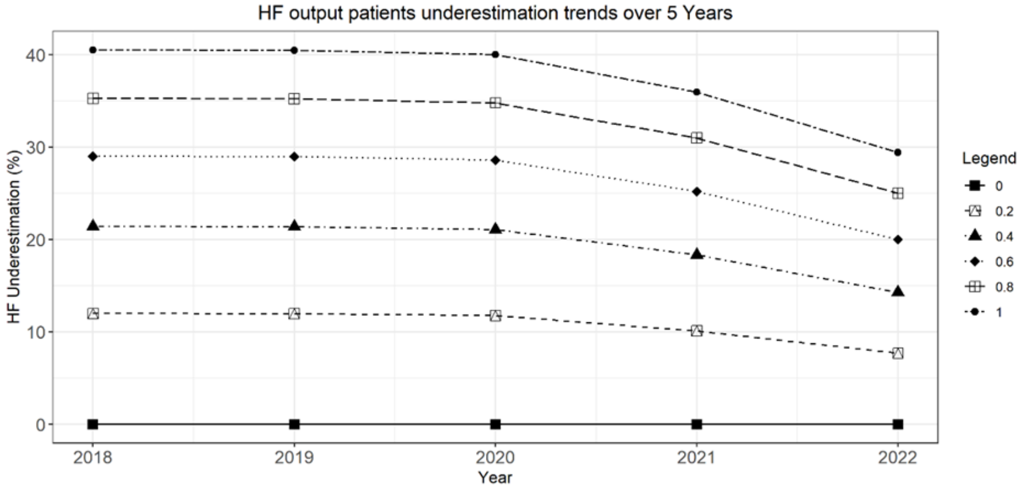

B

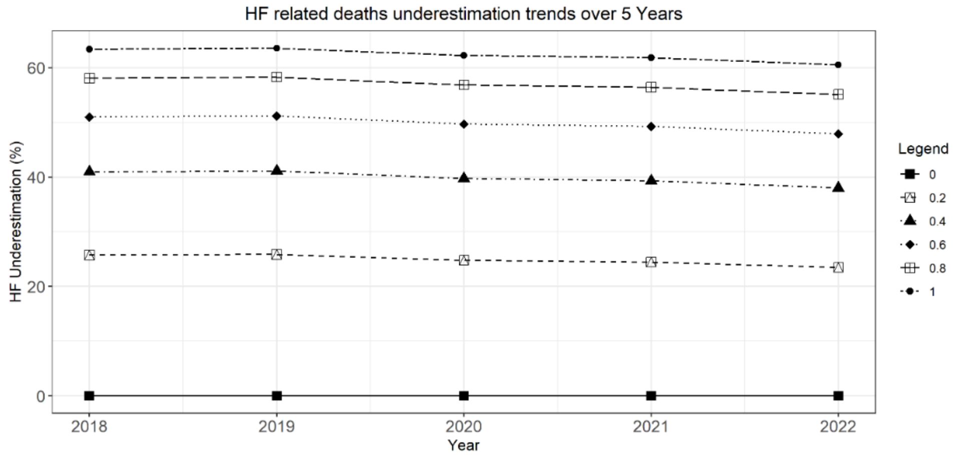

Figure S5. Reliability diagram summarizing calibration across all datasets.

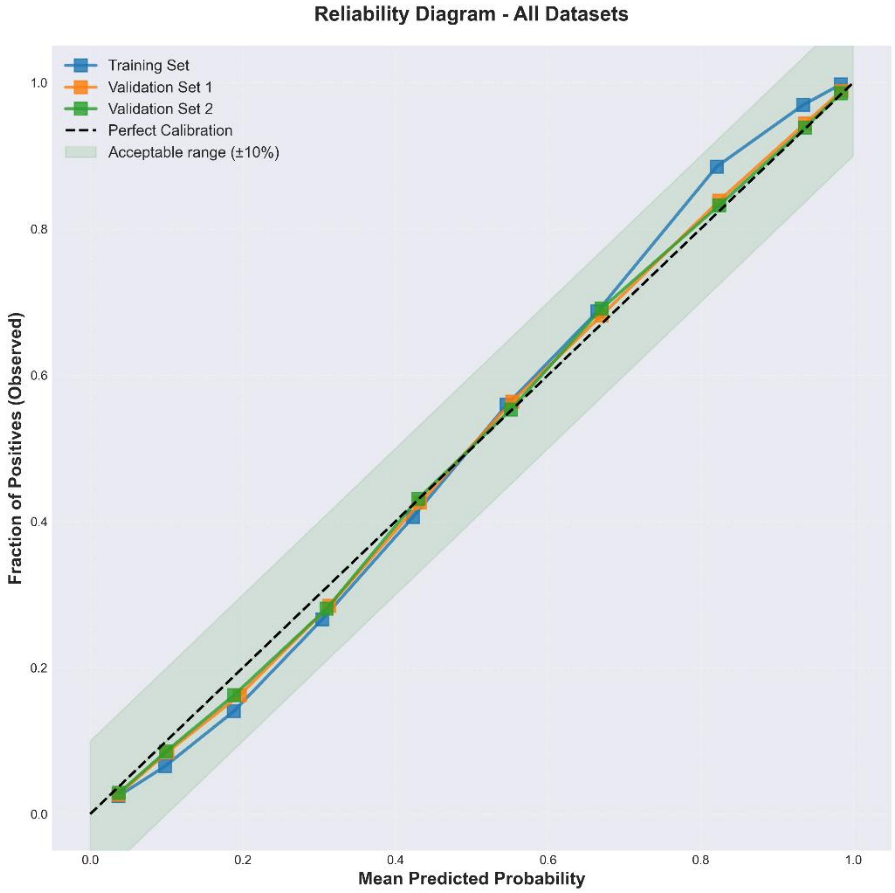

Figure S6. Calibration plots (reliability curves) for the CatBoost model in the training and validation datasets.

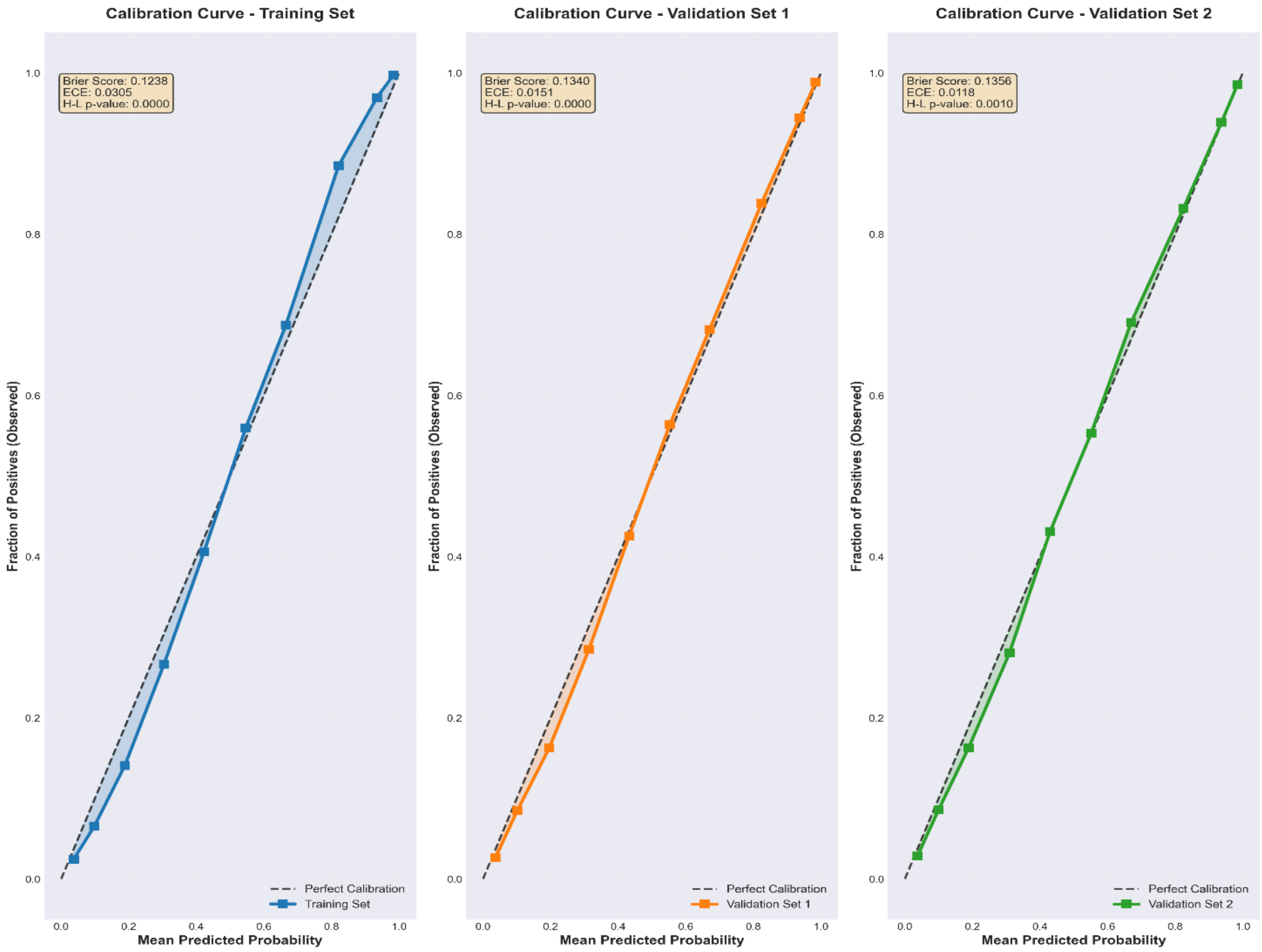

Figure S7. Distribution of SHAP Values and Feature Contributions in the Model. Each feature in the model is represented by specific input values. For ICD codes and procedures, the value indicates the number of times each was recorded in the patient's journey. Race and sex are represented as binary values: 0 for females and 1 for males; 0 for non-black or mixed race and 1 for mixed race. Age and BMI are expressed as integer values (years and kg/m^2). The color scale indicates the magnitude of feature values: blue represents the lowest values, purple signifies medium values, and pink denotes the highest values. Features are presented in order of importance, with the most relevant listed first. On the SHAP axis, negative values indicate a detrimental impact on the prediction, while positive values indicate a beneficial impact. For example, for the transthoracic echocardiogram, fewer occurrences of this procedure suggest a lower likelihood of being predicted as having HF.

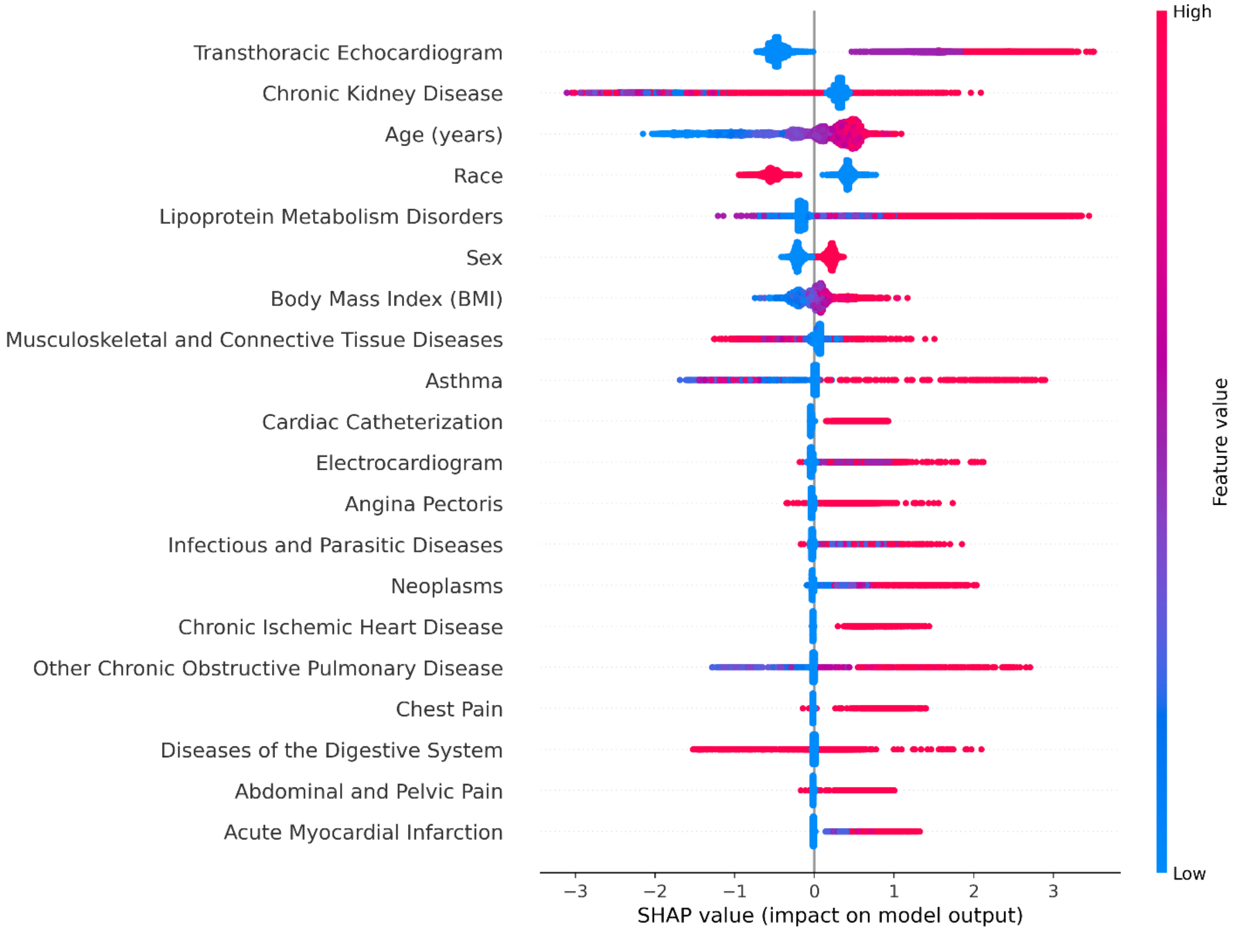

Table S1. Variables Selected for Model Development

Category	Variables
Demographic information	Age
	Age group
	Sex
	Race
	Body Mass (BMI)
ICD Classifications	ICD Chapters (A-D, L, M, O, P, Q, K)
	Specific ICD codes (K7, K8, E1, B57, F52, F32, F33)
	All three-digit ICDs for Chapters E, I, R, J, and N
Medical Procedures and tests	Measurement of natriuretic peptides (BNP and NT-proBNP)
	Stress echocardiography
	Transesophageal echocardiography
	Transthoracic echocardiography
	Cardiac catheterization
	Arterial blood gas analysis (post-exercise cycle ergometry)
	IgG antibody testing for *Trypanosoma cruzi*
	Ambulatory blood pressure monitoring (ABPM)
	Electrocardiogram (ECG)
	Measurement of glycosylated haemoglobin (HbA1c)
	Measurement of aldosterone levels
	Measurement of cortisol levels
	Measurement of catecholamines
	Glomerular filtration rate assessment

Table S2. Parameters and performance for Random Search and Skopt.

Technique	Parameters	Accuracy	AUC	Recall	F1-Score
Random Search	learning_rate:0.046; l2_leaf_reg: 5; iterations:800; depth: 7; border_count:21	0.7829	0.8739	0.7777	0.7820
Skopt	learning_rate:0.055; l2_leaf_reg: 10; iterations:800; depth: 5; border_count:10	0.7782	0.87206	0.7757	0.7779
Grid Search	learning_rate: 0.083 ; l2_leaf_reg: 20,25,30,35 ; iterations:900; depth:8; border_count:20,25,30,35	0.789	0.877	0.779	0.787

AUC - area under the curve

Table S3. Model Metrics Evaluation.

Cross Validation Groups (CV=5)	AUC (mean ± std)	Accuracy (mean ± std)	F1-Score (mean ± std)	Recall (mean ± std)	Brier Score (mean ± std)
Train (N=19,995)	0.877 ± 0.028	0.789 ± 0.026	0.787 ± 0.029	0.779 ± 0.044	0.142 ± 0.017
Validation 1 (N=20,000)	0.879 ± 0.027	0.793 ± 0.027	0.789 ± 0.031	0.777 ± 0.049	0.141 ± 0.017
Validation 2 (N= 17,041)	0.875 ± 0.025	0.788 ± 0.024	0.783 ± 0.029	0.774 ± 0.051	0.144 ± 0.015
**Entire Group**	**AUC**	**Accuracy**	**F1-Score**	**Recall**	**Brier Score**
Train (N=19,995)	0.908	0.822	0.819	0.809	0.124
Validation 1 (N=20,000)	0.891	0.803	0.802	0.798	0.134
Validation 2 (N= 17,041)	0.889	0.802	0.799	0.795	0.135

AUC - area under the curve

Table S4. Logistic regression results and Catboost

Dataset	AUC-ROC	Accuracy	Recall	Precision	F1-Score	Brier Score
Logistic Regression - Training Set	0.837684	0.754139	0.6887	0.79252	0.736972	0.163481
Logistic Regression - Validation Set 2	0.793169	0.708008	0.702857	0.047018	0.08814	0.210922
CatBoost	0.83707	0.664181	0.822857	0.047368	0.08958	0.189411

ROC - Receiver Operating Characteristic; ACU - area under the curve

Table S5. Calibration metrics for the CatBoost model in the training and validation data sets.

**Metric**	**Training Set**	**Validation Set 1**	**Validation Set 2**
Brier Score	0.124	0.134	0.136
Expected Calibration Error (ECE)	0.030	0.015	0.012
Maximum Calibration Error (MCE)	0.080	0.033	0.040
Calibration Slope	1.26	1.10	1.07
Calibration Intercept	0.027	–0.027	–0.026
Hosmer–Lemeshow χ^2 (df = 8)	204.7	43.5	26.1
Hosmer–Lemeshow p-value	<0.001	<0.001	0.001
